# Patient participation in surgical site infection prevention: perceptions of nurses, physicians and patients

**DOI:** 10.1590/1980-220X-REEUSP-2022-0459en

**Published:** 2023-07-24

**Authors:** Mayra de Castro Oliveira, Camila Dalcól, Rhanna Emanuela Fontenele Lima de Carvalho, Vanessa de Brito Poveda

**Affiliations:** 1 Universidade de São Paulo, Escola de Enfermagem, São Paulo, SP, Brazil.; 2 Universidade Estadual do Ceará, Centro de Ciências da Saúde, Fortaleza, CE, Brazil.

**Keywords:** Patient Participation, Surgical Wound Infection, Health Education, Perioperative Nursing, Infection Control, Participación del Paciente, Infección de la Herida Quirúrgica, Educación en Salud, Enfermería Perioperatoria, Control de Infecciones, Participação do Paciente, Infecção da Ferida Cirúrgica, Educação em Saúde, Enfermagem Perioperatória, Controle de Infecções

## Abstract

**Objective:**

To analyze the perception of patients and health professionals regarding patients’ participation in surgical site infection prevention.

**Methods:**

Cross-sectional study conducted in two hospitals in the city of São Paulo, with a convenience sample of 123 patients in the postoperative period of elective surgeries and 92 health professionals (physicians and nurses) acting in direct care of surgical patients.

**Results:**

Patients (78.9%) and professionals (79.4%) fully agreed with the importance of patient participation to prevent surgical site infection. The impact of patient participation on infection rates was significant for those undergoing previous surgery (p = 0.021). Patients and professionals disagreed about the best time to prepare the patient about the topic (p<0.001). The participation strategies considered most effective by patients and professionals were, respectively, oral presentation (47.2% and 75%), videos (40.7% and 58.7%) and leaflets (30.9% and 58.7%).

**Conclusion:**

Patients and health professionals believe that patient participation in surgical site infection prevention is important.

## RESUMO

**Objetivo:**

Analisar a percepção de pacientes e profissionais de saúde sobre a participação dos pacientes na prevenção de infecção do sítio cirúrgico.

**Método:**

Estudo transversal realizado em dois hospitais da cidade de São Paulo, com amostra por conveniência de 123 pacientes no período pós-operatório de cirurgias eletivas e 92 profissionais de saúde (médicos e enfermeiros) atuantes no cuidado direto a pacientes cirúrgicos.

**Resultados:**

Pacientes (78,9%) e profissionais (79,4%) concordaram totalmente com a importância da participação do paciente para a prevenção de infecção do sítio cirúrgico. O impacto da participação do paciente nas taxas de infecção foi significativo para aqueles submetidos à cirurgia prévia (p = 0,021). Pacientes e profissionais discordaram quanto ao melhor momento para preparar o paciente sobre a temática (p<0,001). As estratégias de participação consideradas mais efetivas pelos pacientes e profissionais foram, respectivamente, exposição oral (47,2% e 75%), vídeos (40,7% e 58,7%) e panfletos (30,9% e 58,7%).

**Conclusão:**

Pacientes e profissionais de saúde julgam ser importante a participação do paciente na prevenção de infecção do sítio cirúrgico.

## RESUMEN

**Objetivo:**

Analizar la percepción de pacientes y profesionales de salud sobre la participación de los pacientes en la prevención de la infección del sitio quirúrgico.

**Método:**

Estudio transversal realizado en dos hospitales de la ciudad de São Paulo, con una muestra de conveniencia de 123 pacientes en el postoperatorio de cirugías electivas y 92 profesionales de salud (médicos y enfermeros) que actúan en el cuidado directo de pacientes quirúrgicos.

**Resultados:**

Los pacientes (78,9%) y los profesionales (79,4%) estuvieron totalmente de acuerdo con la importancia de la participación de los pacientes para prevenir la infección del sitio quirúrgico. El impacto de la participación de los pacientes en las tasas de infección fue significativo para los sometidos a cirugía previa (p = 0,021). Pacientes y profesionales discreparon sobre el mejor momento para preparar al paciente sobre el tema (p < 0,001). Las estrategias de participación consideradas más eficaces por pacientes y profesionales fueron, respectivamente, la presentación oral (47,2% y 75%), los vídeos (40,7% y 58,7%) y los folletos (30,9% y 58,7%).

**Conclusión:**

Los pacientes y los profesionales de salud creen que la participación de los pacientes en la prevención de la infección del sitio quirúrgico es importante.

## INTRODUCTION

Surgical site infection (SSI) represents 14**–**16% of health care-associated infections (HAI) being the most prevalent postoperative complication as a result from surgical procedures, affecting up to 20% of patients, which may impact morbidity and mortality, generate physical, psychological and/or social harm, and compromise patients’ safety^(1,2)^. Furthermore, it is associated with a lengthy hospital stay, on average 9.7 days, an 11-fold increase in mortality risk, an increase in the cost of US$ 20,000 per admission, and the possible need for admission to the Intensive Care Unit (ICU)^(2)^.

The diagnosis of SSI is based on the development of signs and symptoms of infection within thirty days after surgery without implants, or ninety days after surgery with implants. Regarding the topography of involvement, it can be classified as: superficial incisional SSI, when it involves skin and subcutaneous tissue; deep incisional SSI, when it affects deep soft tissues, such as fascia and muscle; and organ-space SSI, when it covers the cavity and organs manipulated during surgery^(2)^.

It has been estimated that 60% of SSI would be avoidable through the adoption of orientation and prevention measures^(1)^. Informing, educating, and involving patients in their care is a relatively recent strategy that has been used to prevent and control infections, with positive results in international studies^(3–5)^. A systematic review on patient safety showed that there are several strategies that promote patient participation, such as questionnaires, interviews, focus groups, videos, information leaflets and semi-structured interviews^(6)^.

Patient participation is defined as a process in which the individual holds greater control over the actions and decisions that affect his/her own health, involving the understanding of his/her role, acquisition of knowledge to actively collaborate with the health team, and development of skills that allow such participation^(7)^.

A recent study that investigated the patient’s participation on the safety of care points out that the patient’s involvement is directly related to their co-responsibility, since they must understand the health-disease process, manage the possible risks involved with their health, committing to carry out the guidelines received from health professionals^(8)^.

Thus, in this study, patient participation should be understood as involvement, co-responsibility, understanding of the perioperative process, risk management and commitment to care, listing the patient as a central and active agent in the prevention of SSI.

Although there are several publications on the application, adherence and impact of preventive measures of SSI applied in the perioperative period, there is a lack of international and national scientific production on patient participation in this process and how it affects the rates of surgical site infection. Thus, investigating the perception of patients and health professionals on the subject, considering the national reality, will provide an overview of the most effective strategies to promote this commitment effectively.

Given the above, the following research question arises: What is the perception of patients and health professionals about the patients’ participation in the prevention of SSI? Thus, the aim of this research was to analyze the perception of patients and health professionals about the participation of patients in the prevention of SSI.

## METHOD

### Type of Study

Cross-sectional study characterized by direct observation of a certain number of individuals at a single time, with the advantage of a greater power of generalization and hypothesis building^(9)^.

### Study Site

The study was conducted in two health facilities located in the city of São Paulo - SP - Brazil, namely, a secondary teaching hospital that serves patients through the Unified Health System (SUS), which has 147 beds, 10 operating rooms and performs an average of 260 surgical procedures per month, and a large private institution that has 457 beds, 24 operating rooms and an average of 80 surgical procedures per day. Both institutions perform small, medium and large surgeries, have a hospital infection control committee and do not have a preoperative orientation program in place.

### Sample and Selection Criteria

The study used a convenience sampling of 123 elective surgical patients, in the postoperative period, admitted to the surgical clinic and 92 health professionals (physicians and nurses) involved in the direct care of surgical patients at any time during the perioperative period.

Inpatients older than 18 years old, in the postoperative period of elective surgeries were included, regardless of surgical specialty, and excluded patients in intensive care units, emergency, with cognitive changes or dementia, illiterate or visually impaired.

As for the professionals, we included surgeons and nurses (working in inpatient units and surgical center) involved in the care of surgical patients for a period longer than 12 months. Healthcare professionals who worked only in intensive care unit, emergency department, and outpatient clinic were excluded.

### Sample Definition

Convenience sampling was selected by the researcher, during the data collection period, on a daily basis by checking which inpatients met the inclusion criteria.

The identified patients were approached at the hospital bed, from the first postoperative day, by the researcher who presented the research proposal and asked for their signature of the Informed Consent Form (ICF).

Healthcare professionals were personally approached in their workplace (inpatient units and surgical center) expressing their agreement to participate by signing the ICF. Moreover, they could choose to answer the data collection instrument at the time they deemed most appropriate, either in print, which would be returned to the researcher, or via e-mail.

Emerging doubts in filling out the instruments, among patients or professionals, were specifically addressed by the researcher.

### Data Collection

Data collection was carried out by the main researcher from January 14 to February 24, 2021. To this end, two data collection instruments were constructed, composed of multiple-choice questions, with answers in a five-point Likert scale format, ranging from “strongly disagree” to “strongly agree,” one directed to patients and the other to health professionals, containing the following domains: sociodemographic data (gender, level of education, socioeconomic status, surgery performed and health institution for patients; and professional category, specialization, time of experience and area of practice, for health professionals); perception on patient participation (importance of participation, perception on the impact on reducing SSI rates, desire for participation/implementation in care practice, preparation of professionals); strategies for patient participation (most appropriate time, types of strategies, forms of post-discharge follow-up); patients’ previous experiences with SSI (previous surgeries and SSI); and professionals’ participation in SSI prevention (implementation of strategies in current practice).

The instrument was submitted to face validation and evaluated by five referees, of whom two had a doctoral degree and three a master’s degree, with a mean of eight (±2.77) years of professional experience and expertise in the surgical and infection prevention areas, with relevant publications in perioperative nursing.

The referees showed agreement (Content Validity Index) greater than 90% on the aspects of the instrument and its items, regarding clarity, comprehensiveness and relevance. It was suggested to change the term “surgical site infection” to “surgical wound infection” in the instrument aimed at patients; to adjust the language of some questions to improve laypersons’ understanding; to change the order of some questions to promote better fluidity between the items; and to add the question about which professional provided guidance on surgical wound care. The suggestions made by the referees were included in the instrument aiming to achieve the research objective.

After authorization from the participating institutions and the Research Ethics Committee, a pilot test of the instruments was performed with three participants, who were not included in the study, to verify the suitability and application of the instrument.

### Study Variables

The *dependent variables* addressed were the perception of patients and healthcare professionals about the importance of patient participation in surgical site infection prevention and the potential impact on reducing SSI rates. The *independent variables* for patients were: gender; age in years; level of education; socioeconomic status in number of monthly minimum wages; surgery the patient underwent; public or private institution where the surgery was performed. For the practitioners, the following variables were evaluated: gender; age in years; professional group (Nurse or Physician); area of specialization; professional experience in years; public or private work institution.

### Data Analysis and Treatment

Data were tabulated in Excel spreadsheet and analyzed using the *Statistical Package for Social Sciences* (SPSS) 21.0 software.

The following analyses were used: categorical variables were assessed using the chi-square test and Fisher’s exact test; comparison between numerical variables was performed using the Kruskal-Wallis, Wilcoxon-Mann-Whitney, Student’s and Welch’s t-tests and ANOVA model; ordinal logistic regression was used to find the predictors of the variables: importance of patient participation and desire to be involved in SSI prevention. The significance level adopted was p = 0.05.

### Ethical Aspects

The project was cleared by the Research Ethics Committee of the School of Nursing of the University of São Paulo under opinion no. 4,362,054 and CAAE no. 37225720.1.0000.5392, after authorization from the institutions selected for this study, based on Resolution no. 466 of December 12, 2012.

All patients and health practitioners were approached and informed about the content and objectives of the research, expressing their agreement to participate in the investigation by signing the ICF before the beginning of the research, in two copies, one of which was given to the participant and the other remained in the possession of the researcher.

## RESULTS

Among the 123 patients sampled, 52% were male, with high school education (33.3%) or completed postgraduate studies (25.2%), who underwent mainly general surgery (44.7%), from private institutions (56.9%), mean age 47 (±15) years and monthly income of 14 minimum wages, 78% had performed some type of surgery previously and of these, only 7.3% had SSI in previous surgical experiences.

Regarding practitioners, 92 people participated in the study, with mean age of 45.9 (±10.7) years, 56.5% were male, 57.6% were physicians, 64.1% worked in the OR, 79.3% came from private institutions and had a mean of 19.3 years of professional experience.

Most patients and professionals, respectively, totally agreed (78.9% and 79.4%) with the importance of patient participation for the prevention of SSI, and how this participation could result in a possible impact on reducing SSI rates (75.6% and 75%) and professional preparation for the inclusion of patients in SSI prevention actions (73.2% and 60.9%), pointing no statistically significant difference.

Table 1 shows that being men reduces the chance of agreeing with the desire to be involved in SSI prevention by 67% (p = 0.043). Although the other associations were not statistically significant, the following aspects stand out: income increases the degree of agreement with the statements and patients seen at public hospitals and with lower educational level are less likely to agree with the highlighted statements.

**Table 1:** Associations between demographic, socioeconomic and surgical variables of patients with the level of importance of patient participation and desire for involvement in the prevention of SSI – São Paulo, SP, Brazil, 2021.

Variables	Importance of patient participation			Desire for involvement in SSI prevention		
	OR	CI 95%	p-value*	OR	CI 95%	p-value*
**Age**	1,01	[0,97; 1,06]	0,542	1,01	[0,97; 1,05]	0,718
**Men**	0,50	[0,15; 1,59]	0,245	0,33	[0,11; 0,94]	0,043
**Schooling**						
Basic Schooling	8,52	[0,41; 300,50]	0,179	14,25	[1,00; 426,04]	0,069
High School	3,24	[0,23; 45,02]	0,372	3,58	[0,41; 32,62]	0,248
College	1,60	[0,07; 36,46]	0,767	1,64	[0,13; 21,72]	0,705
Postgraduate	1,73	[0,06; 47,63]	0,745	1,35	[0,08; 22,83]	0,836
**Income**	1,01	[0,98; 1,05]	0,575	1,02	[0,99; 1,07]	0,237
**Previous Surgery**	1,82	[0,46; 6,89]	0,380	0,65	[0,17; 2,24]	0,507
**Public hospital**	0,72	[0,10; 4,43]	0,732	0,35	[0,06; 1,68]	0,206

*OR: odds ratio; CI: Confidence Interval SSI: surgical site infection;* *Ordinal Logistic Regression.

Table 2 shows the perception of health practitioners regarding the importance of patient participation and the impact of their involvement in the prevention of SSI, that each additional year of age reduces the degree of agreement with the statements (respectively, 9% and 11%), that physicians are less likely to agree with the statements (respectively, 47% and 13%), as well as professionals who work in the public service (respectively, 58% and 36%). On the other hand, each additional year of professional experience increases the chance of agreement (respectively, 6.0% and 8.0%).

**Table 2:** Associations of demographic, socioeconomic and work variables of professionals with the level of importance of patient participation and impact of patient involvement in the prevention of SSI – São Paulo, SP, Brazil, 2021.

Variables	Importance of patient participation			Impact of the involvement in SSI prevention		
	OR	CI 95%	p-value*	OR	CI 95%	p-value*
**Age**	0,91	[0,79; 1,04]	0,154	0,89	[0,79; 1,00]	0,060
**Men**	1,82	[0,28; 11,19]	0,520	2,27	[0,46; 11,58]	0,314
**Professional group (physician)**	0,53	[0,06; 4,52]	0,560	0,87	[0,13; 6,05]	0,884
**Length of professional experience**	1,06	[0,93; 1,21]	0,364	1,08	[0,96; 1,21]	0,218
**Public hospital**	0,42	[0,13; 1,43]	0,150	0,64	[0,20; 2,32]	0,474
**Implementing a strategy for preventing SSI**	0,90	[0,18; 3,51]	0,887	0,38	[0,05; 1,65]	0,246

OR: odds ratio; CI: Confidence Interval SSI: surgical site infection; *Ordinal Logistic Regression.

Only 5.7% of patients totally disagreed about the reduction in SSI rates by participating in preventing this complication, in contrast, the patient having previously undergone surgery was associated with the perception that patient participation impacts SSI rates (p = 0.021). The variables age (p = 0.402), gender (p = 0.308) and level of education (p = 0.630) were not associated with the perception that patient participation impacts on SSI rates. As for the professionals, age (p = 0.276), time of experience (p = 0.446) and professional category (p = 0.087) were not associated with the perception that patient participation impacts on SSI rates.

Regarding professional preparation for the inclusion of patients in SSI prevention, 96.7% of health professionals agreed with its importance, 88% would like to implement it in their care practice, 77.1% believe that the work process would favor this action and 85.9% would receive institutional support to implement a program of this nature, and 88.1% realize the need for training of the care team on how to involve the patient in preventing this infection.

Regarding the most appropriate time for patient participation in the prevention of SSI there was divergence between groups (p < 0.001), because health professionals indicated the preoperative period and patients the postoperative period.

Regarding the strategies for patient participation in the prevention of SSI, there is a significant difference between the perception of health professionals and patients about the use of oral presentation (p < 0.001), videos (p = 0.009) and leaflets (p < 0.001). For patients, the higher the level of education, the greater the interest in the oral presentation strategy (p = 0.026), and the lower the level of education, the greater the preference for the roundtable (p = 0.033).

For the healthcare professionals, those who work in public institutions showed more interest in the roundtable strategy (p = 0.032). Regarding the professional category, nurses preferred leaflets (76.9%), oral presentation (74.4%) and video (59.0%), while physicians preferred oral presentation (75.5%), video (58.5%) and leaflets (45.3%).

Regarding the implementation of SSI prevention strategies, 82.6% of health professionals reported that they already do it and apply it preoperatively (62.0%), intraoperatively (28.3%) or postoperatively (56.5%), and they mainly use oral presentations (76.1%), leaflets (20.7%) and videos (15.2%) (Table 3).

**Table 3:** Distribution of patients and health practitioners according to preference for patient participation strategies for the prevention of SSI and forms of post-discharge follow-up – São Paulo, SP, Brazil, 2021.

Variables	Patients (n = 123) n (%)	Health practitioners (n = 92) n (%)	p-value*
**Participation Strategies**			
Oral Presentation	58 (47,2)	69 (75,0)	<0,001
Video	50 (40,7)	54 (58,7)	0,009
Leaflets	38 (30,9)	54 (58,7)	<0,001
Roundtables	22 (17,9)	11 (12,0)	0,234
Focus groups	4 (3,3)	7 (7,6)	0,152
Incident reporting	4 (3,3)	6 (6,6)	0,261
Others†	3 (2,4)	2 (2,2)	0,899
**Post-discharge follow-up**			
WhatsApp® message	66 (53,7)	44 (47,8)	0,398
Videocall	51 (41,5)	20 (21,7)	0,002
Outpatient follow-up	39 (31,7)	61 (66,3)	<0,001
Phone contact	27 (22,0)	32 (34,8)	0,037
Cell phone app	26 (21,1)	38 (41,3)	0,001

SSI: surgical site infection; *Pearson’s X ^2^;

† Other: explanatory materials, SMS, e-mail, and office dialogue.

The fact of implementing SSI prevention strategies is not related to the perception of greater or lesser importance of patient participation (p = 0.698). However, there is an association between who implements strategies postoperatively and the perceived importance of patient participation (p = 0.037).

About the best form of follow-up after discharge from SSI, 66.3% of professionals considered the outpatient return and 53.7% of patients indicated messages by WhatsApp®. There is a significant difference between the perception of the best post-discharge follow-up strategies by health professionals and patients, namely cell phone app (p = 0.001), outpatient follow-up (p < 0.001), telephone contact (p = 0.037) and video call (p = 0.002) (Table 3).

Patients with lower levels of education (p < 0.001) and from public institutions (p < 0.001), as well as professionals from public institutions (p < 0.001), prefer outpatient follow-up after discharge, while professionals (p = 0.019) and patients (p = 0.019) from private institutions preferred the use of WhatsApp® messages. The use of telephone contact as a form of post-discharge follow-up was divergent between physicians and nurses (p = 0.001). In addition, older patients preferred the use of telephone (p = 0.008), while younger patients preferred video call (p = 0.001).

## DISCUSSION

Patient participation is currently receiving growing attention in the healthcare field, especially participation in the prevention and control of hospital-acquired infections, especially SSI. Encouraging patients to participate in their care and safety is the focus of ongoing efforts in several countries and medical systems^(10)^.

The present analysis revealed that patients and healthcare professionals agree on the importance of patient participation in preventing SSI. However, they may have different views on what patient participation is, so it seems important to emphasize that patient and health care professional should agree upon a common appreciation of what is meaningful to support patient participation in health care^(11)^.

Perioperative nursing care has changed from a problem-centered task to a patient-centered attitude, which reveals efforts and changes involving research and implementation of evidence-based practices. Patient participation in the prevention and control of SSI requires a change from the role of “receiver” to the role of “collaborator” in this process, with a significant impact on their intention to participate in the prevention and control of this complication^(10)^.

Concurrent with the present study, Yao et al.^(10)^ demonstrated that the medical team supports patient participation and is willing to actively communicate with the patient in order to inform the patient, clarify safety issues and meet needs to achieve better outcomes. It is worth noting that, trust between healthcare professional and patient impacted patient participation in the control of SSI, being an important factor of influence and patient satisfaction. Establishing a relationship of mutual trust during the perioperative period may increase patients’ compliance with their behavior towards the prevention and control of SSI^(10)^.

A study conducted in China with 580 surgical patients showed that patients have a high level of intention to participate in the prevention and control of SSI. However, the actual behavior was lower than the intention, being affected by various factors such as motivation, self-efficacy, medical and social support. Therefore, it is necessary that the healthcare team is prepared to maximize the patient’s desire to participate in the process, by involving family members as a social support system and promoting support and communication, factors that positively affect the patient’s involvement and participation^(10)^.

In the present study, patients from public institutions were less likely to agree with the importance of participation and desire to be involved in the prevention of SSI, as well as lower income and education level, which may be associated with less access to information. In addition, professionals from public institutions are also less likely to agree with the importance of patient participation and its impact on preventing SSI.

A similar study highlights social determinants, the culture of medicalization, the care model, the organizational support and professional training as influencing the patients’ involvement with their care. The social determinants can affect the patient’s commitment to the orientations received, highlighting mainly the level of education, which affects the patient’s understanding of health care^(8)^. Patients with low health literacy are less likely to ask questions to health professionals, because they are concerned about what they may think about their ability to understand the disease and the information about the treatment^(10)^.

In the present study, practitioners showed interest in involving patients as agents of SSI prevention, believe they have institutional support and that the activities are compatible with their workload; however, they emphasize the importance of institutional training for the care team to develop this process of patient inclusion in their care.

On the contrary, practitioners from a hospital in Sweden evidenced that the health team does not consider care as the patient’s responsibility and, for this reason, they do not provide opportunities for patients to express their choices. The patient is seen as passive, rarely questioning the care offered, and patients may occasionally present resistance to guidelines and make it difficult for them to participate in the prevention of HAI. The study points out that the organization and the healthcare professionals are not sufficiently prepared to involve the patient in HAI prevention^(12)^.

Health practitioners approached in another study understand that organizational support can positively or negatively influence the patients’ involvement in their care, for example, often the lack of time and/or the number of professionals on the team do not allow patient education. Nevertheless, they believe that the patient’s participation is more linked to the attitude of the practitioners than to the institutional culture itself^(8)^.

A study that assessed the nursing team’s knowledge about pre- and trans-operative care for the prevention of SSI showed satisfactory knowledge of professionals (80% to 100%), and also pointed out the need for constant improvement of professionals in order to minimize the knowledge deficit as the subject is updated^(13)^.

The training of the multiprofessional team should start since the academic training, and remain through workshops and training courses, besides that, the professional experience itself favors the knowledge and safety for patient education^(8)^. Health education on SSI prevention and control should be strengthened by health professionals and the patient should be empowered for such function, considering him/her as a cooperative and capable partner^(10)^.

Surgical patients believe that the best time to be prepared for the prevention of SSI is the postoperative period, while health professionals argue that the best time is preoperative. Despite the clarity about the importance of patient participation in this context, there is still a lack of literature on the study of educational materials, their effect on the occurrence of SSI and the best time for their application. A review study that evaluated the effectiveness of the intervention with educational materials for surgical patients showed that there was a positive evaluation by the patients; however, it was not possible to establish its effectiveness and the best time for its application, that is, whether before or after the surgical procedure^(14)^.

Regarding patient participation strategies, it was found that both health professionals and patients have the same preferences: oral presentations, videos, and leaflets, in that order. The scientific community has produced several studies that aim to identify and evaluate the application of certain strategies, whether isolated or not^(4,14,15,16,17)^.

Patient education strategies include clear and simple language between patients and practitioners, use of printed materials as informative means, and audiovisual resources in order to facilitate understanding; and include patients with lower levels of education. Besides these, groups to exchange experiences among patients, relatives, and professionals is also a positive strategy, highlighting the protagonism of the patient and the involvement of the family as a key component in this process^(8)^.

A review study shows that nurses (77.14%) stand out as the main professional to implement education strategies for surgical patients, followed by physicians (34.29%). The most used education resources were the use of booklets with text and images, videos and educational leaflets, and as the main strategy, traditional education, with verbal guidance and solving doubts^(17)^.

Intra-hospital and post-discharge follow-up of SSI requires active, patient-based surveillance with information on signs and symptoms within the surveillance period. Surveillance strategies after discharge include contact with the patient by mail, telephone, a combination of these methods, or any other method with the ability to identify possible SSI, according to NHSN criteria^(2)^.

It is worth noting the use of mobile technology as a post-discharge surveillance strategy for SSI, which has shown promise by national and international studies^(18–20)^. A review study evidenced the importance of technology in patient education about self-management of health and remote monitoring by health professionals, showing that patients are willing to use mobile technologies and *mHealth* applications^(18)^.

An USA study pointed out that the monitoring of the surgical wound by means of mobile technology using images, showed a high level of fidelity in the identification of SSI, user satisfaction and willingness of patients and caregivers to use it, with an overall response rate of 90.2%^(19)^.

In the same vein, a randomized clinical trial showed that patients who used mobile technology had a 3.7 times greater chance of a diagnosis of SSI in the first seven postoperative days, reduced the frequency of hospital care and improved the experience in access to care, concluding that remote monitoring of the surgical wound facilitates the triage and promotes early diagnosis of SSI^(20)^.

Given the evidence presented in this study, regarding patient participation in the prevention of SSI, it seems that there is a need for alignment between the wishes of patients and the perceptions of healthcare professionals. Moreover, the patients’ answers seem to reveal that the post-discharge follow-up strategies deserve to be reviewed, serving as a warning to professionals when designing their healthcare interventions.

Regarding limitations, the study was composed of a convenience sample, which may limit the generalizability of the data; patients were approached only in the postoperative period and the collection instruments, produced by the authors, were subjected only to face validation.

## CONCLUSION

It was concluded that patients and health professionals consider important the participation of patients as active members of their care in the prevention of SSI. For this, strategies such as oral presentations, videos and pamphlets can be used for patient education in the pre- and postoperative periods, with the training of healthcare professionals being indispensable.

It is expected that the results of this study will help health professionals to identify the best strategies for the participation, involvement and commitment of patients as active subjects in their care, regarding the prevention of SSI. Future investigations with patients, families and healthcare professionals, development of innovative strategies for patient empowerment, and comparison of education in the preoperative and postoperative periods are suggested.
